# Supporting informed clinical trial decisions: Results from a randomized controlled trial evaluating a digital decision support tool for those with intellectual disability

**DOI:** 10.1371/journal.pone.0223801

**Published:** 2019-10-23

**Authors:** Lauren A. McCormack, Amanda Wylie, Rebecca Moultrie, Robert D. Furberg, Anne C. Wheeler, Katherine Treiman, Donald B. Bailey, Melissa Raspa

**Affiliations:** 1 Public Health Research Division, RTI International, Research Triangle Park, North Carolina, United States of America; 2 Center for Newborn Screening, RTI International, Research Triangle Park, North Carolina, United States of America; 3 Health Quality & Analytics, RTI International, Research Triangle Park, North Carolina, United States of America; Radboud University, NETHERLANDS

## Abstract

**Background:**

Informed consent requires that individuals understand the nature of the study, risks and benefits of participation. Individuals with intellectual disabilities (ID) have cognitive and adaptive impairments that may affect their ability to provide informed consent. New treatments and clinical trials for fragile X syndrome, the most commonly known inherited cause of ID, necessitate the development of methods to improve the informed consent process. The goal of this study was to compare the efficacy of a digital decision support tool with that of standard practice for informed consent and to examine whether the tool can improve decisional capacity for higher functioning individuals.

**Methods:**

Participants (N = 89; mean age = 21.2 years) were allocated to the experimental group (consenting information provided via the digital decision support tool), or the comparison group (information provided via standard practice). Participants were assessed on four aspects of decisional capacity (Understanding, Appreciating, Reasoning, and Expressing a choice). We used regression analyses to test the impact of the tool on each outcome, repeating the analyses on the higher functioning subsample.

**Results:**

No differences existed in any domain of decisional capacity for the sample in full. However, participants in the higher IQ subsample who used the tool scored better on Understanding after adjustment (β = 0.25, p = 0.04), but not on Appreciating or Reasoning. No differences by experimental group existed in the decision to join the hypothetical trial for the full sample or higher functioning subsample.

**Conclusions:**

A decision support tool shows promise for individuals with fragile X syndrome with higher cognitive abilities. Future studies should examine the level of cognitive ability needed for sufficient understanding, whether these findings can be translated to other clinical populations, and the impact of the tool in larger trials and on trial retention.

## Introduction

Clinical studies with human subjects must follow ethical and legal standards to protect the rights of research participants [1]. Study investigators must provide clear, concise information about the study to potential participants and determine whether an individual is able to make an informed decision. For investigators who study individuals with intellectual disability (ID), the informed consent process may require special modifications or supports to maximize participation in decision making. Fulfilling this obligation in a responsible way requires an understanding of the necessary components of consent, knowledge of individual’s ID, and use of evidence-based adaptations. Although research on recommended informed consent practices is growing, limited data exists about what types of supports are effective for individuals with ID. This study expands the knowledge base through an assessment of an informed consent decision aid used with adolescents and adults with fragile X syndrome (FXS), the most common inherited form of ID. FXS is an excellent prototype because of the wide range of cognitive abilities in affected individuals and the recent increase in clinical trials for new medications targeted at the core biology of FXS.

### Informed consent

In the United States, researchers follow the “Common Rule,” the federal policy that outlines the importance of informed consent. The policy requires researchers to “(1) Disclose information to potential research subjects needed to make an informed decision; (2) Facilitate the understanding of what has been disclosed, and (3) Promote the voluntariness of the decision about whether to participate in the research” [2, 3].

Despite federal requirements about informed consent, variability remains in the ways informed consent is conceptualized and operationalized [4–6], leading to significant confusion and poor implementation. Informed consent should be an *active process* of exchanging information with appropriate supports, enabling a person to decide whether the benefits outweigh the risks for them as an individual [2, 7]. However, traditional informed consent often falls short of best practices in several areas. The communication process is often not bidirectional or tailored to an individual’s needs. Participants can feel overwhelmed with the volume and complexity of information in consent forms, and many report feeling pressured to sign forms before fully reading them [8]. They also frequently misunderstand key components of the study, which can result in consent that is not truly informed [9] and can lead to lower study retention rates [10].

In addition to the process of obtaining informed consent, there is a need to document how engaged a participant is in the process and whether he/she has the ability to make informed decisions about participation. The concept of “decisional capacity” has been proposed as a measure of participant’s decision making abilities and includes four elements: (1) Understanding—perceiving and retaining information; (2) Appreciation—linking the decision to one’s own situation; (3) Reasoning—considering all the information and weighing the consequences, and (4) Making and communicating a choice—reaching and communicating a decision [11].

### Informed consent decision aids

A 2014 systematic review of the literature examining the adequacy of patient-reported measures of informed consent identified a narrow focus on understanding [5]. Consequently, efforts to date to improve the consent process have focused on improving understanding, primarily via changes to the consent forms or use of multimedia interventions. An earlier systematic review [12] found that these approaches had limited success in terms of improving understanding and concluded that a human educator may be most effective. However, the authors noted that the potential of multimedia interventions has not likely been realized [12]. In more recent studies, Beskow [13] and Grady [14] found that comprehension was not inferior among individuals who received a simplified consent form compared with a traditional informed consent group. Kim & Kim [15] found that a simplified consent form that contained plain language, short sentences, diagrams, pictures, and bullet points was associated with higher levels of understanding regardless of health literacy level. A randomized controlled trial comparing a digital consent that combined a video, standard consent language and an interactive quiz to paper consent found that interactive consent improved understanding of study procedures and the risks of a chemotherapy trial [16]. Another randomized control study assessed three multimedia e-educational aids compared to standard text aids to understand medical practice research [17]. Dual-channel approaches, such as animated videos and slideshows with voiceover, were significantly more effective than single-channel techniques in achieving participant understanding of the research.

A sizable body of literature indicates that those who use decision aids when facing a range of treatment or screening decisions feel more knowledgeable, better informed, and clearer about their values [18]. Patients also have more accurate risk perceptions when decision aids are used, either within or in preparation for the clinical consultation [18]. Digital decision support tools have been identified as a strategy to potentially improve the clinical trial consent process through enhanced patient engagement and interactivity [19, 20]. However, according to a 2014 systematic review, it is unclear whether audio-visual interventions can enhance the informed consent process for people considering participating in clinical trials, although trends are emerging with regard to improvements in knowledge and satisfaction [20]. The review demonstrated that considerable heterogeneity exists in terms of the types of audio-visual presentations that have been developed and tested, ranging from “simple audio-visual interventions, such as non-interactive videos, viewed independently, to computer programs with quizzes and hyperlinks, viewed under supervision” ([20], p. 36).

### Informed consent for individuals with intellectual disability

Individuals with ID have cognitive and adaptive impairments that may affect their ability to make informed choices about both routine and significant decisions, such as informed consent. Historical assumptions about impaired decisional capacity often meant that individuals with ID played a minimal role in life decisions [21]. Throughout the last half of the 20th century, the prevailing views have changed as people with ID, disability rights advocates, parents, and ethicists began calling for respect of autonomy and empowerment of individuals with ID, invoking principles like self-advocacy, self-determination, normalization, and opportunity [22].

Clinicians and researchers who study individuals with ID must determine whether a potential study participant is able to make an informed decision and what supports are needed to maximize participation [23]. Studies assessing the capacity to give informed consent of adults with ID have shown promise; one study found that most adults with ID were able to describe the study purpose, their role in the study, voluntary participation, confidentiality of data, and study withdrawal [24]. Another study found that individuals with mild ID have less difficulty understanding, appreciating (i.e., recognizing the impact of research participation on their own care), and reasoning about a hypothetical trial than those with moderate ID [25]. The authors found that an individual’s degree of cognitive disability predicted their total understanding and appreciation and concluded that consent assessment should be individualized for adults with ID for randomized controlled trials (RCTs), even when capacity to give fully informed consent is questionable. Yet another study found that all individuals with ID were able to express a choice about participation, but only 6% were able to meet all requirements of providing informed consent that were put forth by the investigators; these requirements included indicating their choice, understanding the impact, options, risks and benefits, procedures of the study, and nature of the project [26].

In this study, we focus on individuals with the most commonly known inherited cause of ID, fragile X syndrome (FXS). Males with FXS typically have moderate ID, although impairment can range from mild to severe; females typically have mild ID, ranging from normal cognition to moderate impairment [27–29]. Studies have shown cognitive deficits in such areas as sustained attention, response inhibition, working memory, and other executive functions, which are likely to be associated with decisional capacity [30–35]. Individuals with FXS often have a range of co-occurring conditions, the most common of which are attention problems, anxiety, and autism [27, 36].

Advances in understanding the molecular basis of FXS have led to a new generation of treatments and subsequent clinical trials [37]. Although some individuals with FXS likely have a legal guardian who provides consent to treatment on their behalf, many others do not. Regardless of legal guardianship status, all individuals who are asked to take part in clinical research studies should be part of the decision-making process. However, little is known about the extent to which individuals with FXS are involved in decision making about participating in clinical trials. In a study of parents’ perceptions of their adult children’s ability to provide informed consent to participate in FXS clinical trials, only 29% and 7% of parents of male and female children, respectively, rated their adult child as “not at all capable,” with the remaining parents reporting that their adult children could participate in at least some aspects of decision making [34].

### Research objectives

This study had two aims. In the first aim, we examined the extent to which participants with FXS display decisional capacity for informed consent and identified factors associated with decisional capacity. This work was done using paper-based consent materials with the written text read aloud by a research assistant. In analyses reported elsewhere, we found that working memory, verbal memory, and oral presentation of information were significant predictors of understanding scores and that study participants were more likely to understand concrete elements of the trial, such as what they would need to do, but still struggled with abstract concepts such as randomization [38]. In the second aim, we examined the impact of a multimedia decision support tool for individuals with FXS who were deciding whether to participate in a hypothetical clinical trial. We hypothesized that individuals with higher cognitive functioning would have the decisional capacity to make their own decision about participation in clinical trials if they have the appropriate supports. In this paper, we report on findings associated with the second aim addressing three research questions:

1. Compared to standard practice, does the decision support tool improve the decisional capacity of individuals with FXS to make an informed decision about participating in a clinical trial?
2. Do individuals with higher levels of cognitive functioning who are exposed to the decision support tool have greater decisional capacity compared to those receiving standard practice, and if so, in what specific areas?
3. Do higher levels of understanding affect other elements of decisional capacity, namely appreciation, reasoning and the ability to express a choice among individuals with higher levels of cognitive functioning?

## Materials and methods

### Trial design

We used a two-arm RCT design with a 1:1 allocation ratio to examine the efficacy of the decision support tool. We randomized study participants into two conditions: (1) Experimental—exposure to the informed consent information using the decision support tool, and (2) Comparison—standard practice for obtaining informed consent using paper and pencil. No changes were made to the design after the trial commenced.

### Intervention

**Fig 1:**
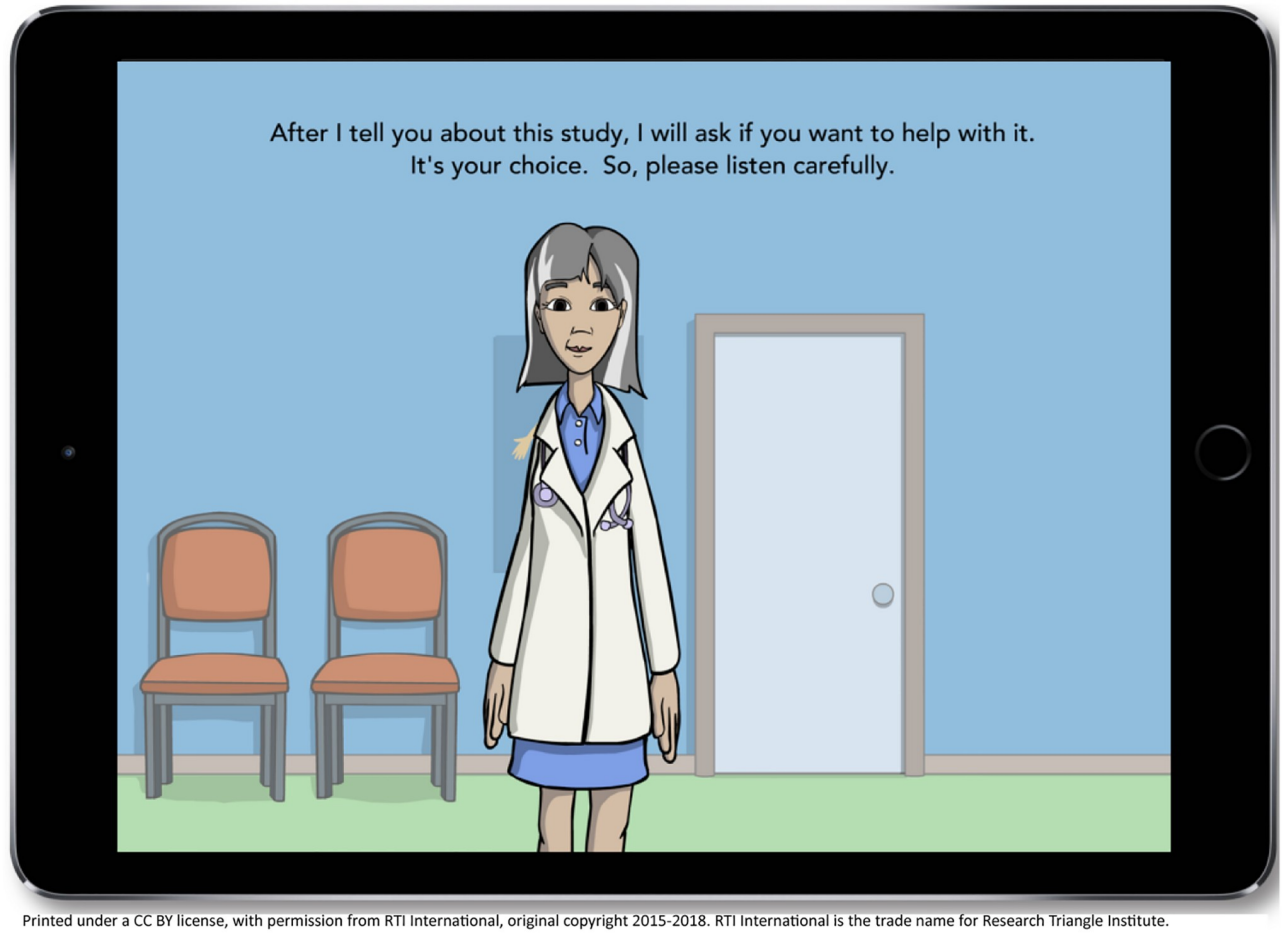
A human-like avatar walks the potential participant through the consent process.

**Fig 2:**
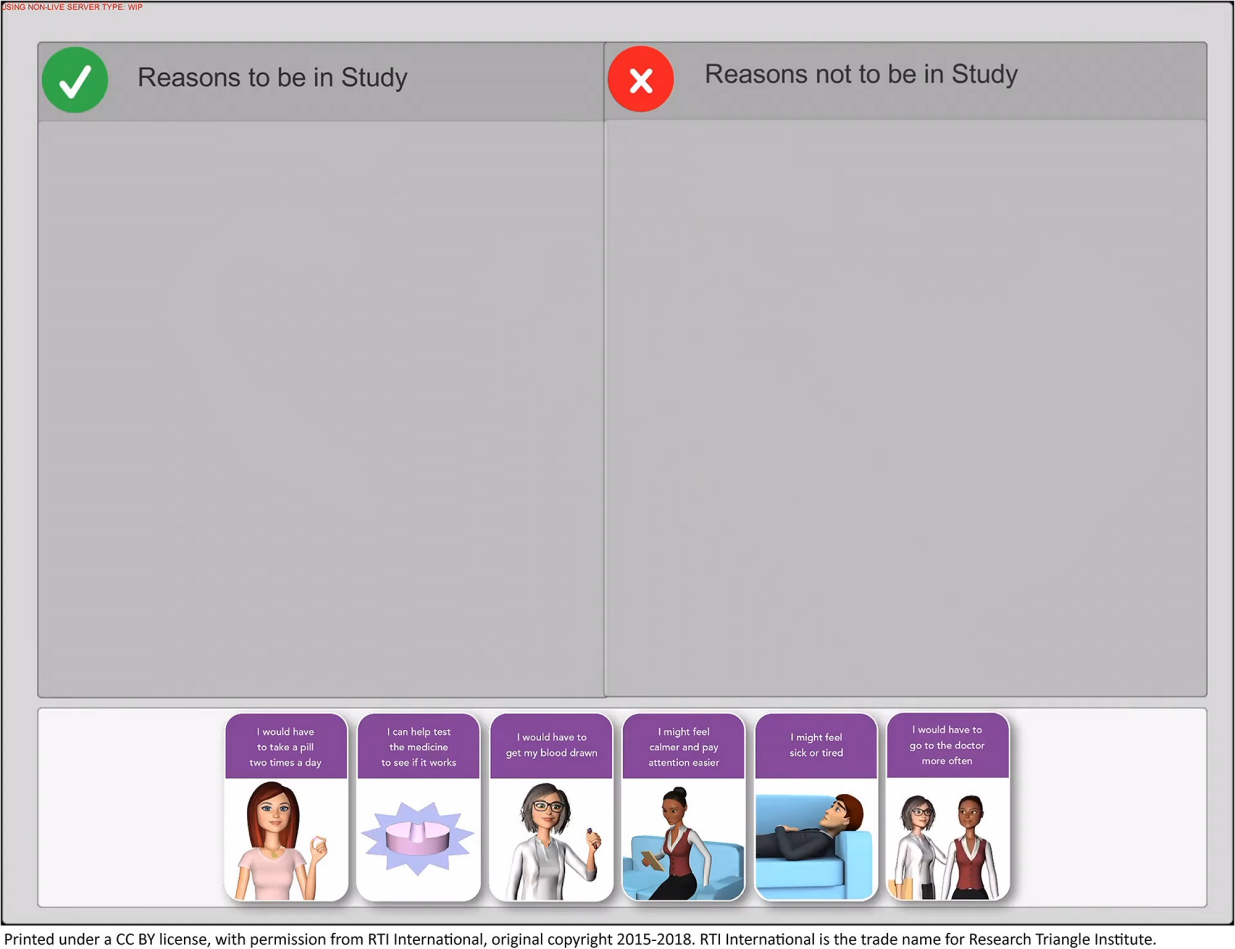
Potential participants engage in a sorting exercise to help articulate their values and preferences.

The content of the standard paper consent form and the decision support tool was based on a protocol for an actual FXS trial even though the trial in the current study was hypothetical. Both the paper form and the digital tool comprise six modules, each including content required for informed consent: the purpose of the trial, components of the trial, how the trial works, benefits of participation, risks of participation, and ability to withdraw. We developed the decision support tool using interactive multimedia—audio, video, and narration—which are delivered on a tablet. A human-like avatar walks the potential participant through the consent process (Fig 1), then the potential participant engages in a sorting exercise with motion graphics (Fig 2) and a knowledge assessment. The sorting activity allows the potential participant to select individual reasons to be or not be in the trial, thereby clarifying an individual’s preferences and values.

The multiple-choice questions in the knowledge assessment each have only one correct answer and three incorrect responses. The tool employs a patient-centered approach rooted in self-determination theory [39] and applies best practices in health literacy and communication science including the use of plain language, digital animations, and effective risk communication strategies to explain complicated aspects of the trial (see Furberg et al. [40] for a detailed description of the design and development process).

### Data collection procedures

As noted earlier, participants were part of a larger study (N = 152) to assess the decisional capacity of individuals with FXS. We collected data during two visits to each participant’s home. During the first visit, we conducted cognitive assessments to use as independent measures in the analyses. We excluded people from the second visit if they would be unable to actively participate because of very low cognitive functioning or other behavioral challenges. Specifically, participants were ineligible if they had an IQ of 30 or less on the Stanford-Binet Intelligence Scales- Fifth Edition (SB-5) using the z-score transformation [41], had an IQ score of 31 to 40 and a diagnosis of autism, or were unwilling to cooperate because of behavioral challenges (e.g., severe aggression, significant attention deficits). These cognitive deficits or behavioral challenges were considered too restrictive for active participation in the hypothetical trial.

Before the second study visit, we mailed all participants a paper consent form describing the hypothetical trial. During the second study visit, for participants in the comparison condition, a research team member used a standardized script to verbally review key areas of the paper consent form. In the experimental condition, participants used the digital decision support tool but also had exposure to the paper consent form. All participants then answered a series of multiple-choice questions (on paper for the comparison group and programmed into the tool for the experimental group) to assess decisional capacity using the measures described in the next section. The study visits took approximately 60–90 minutes each.

We collected the data in 2016 and 2017 after obtaining institutional review board approval from the University of North Carolina at Chapel Hill which served as the IRB of record. Parents of adolescents under the age of 18 provided written permission for their son or daughter to take part in the study. Adolescent participants provided written assent. A similar procedure was followed for adult males participants given that the IRB considered them to be decisionally impaired; parents provided written consent and adult males gave written assent. Adult females who did not have a legal guardian provided their own consent. Otherwise, adult females provided written assent and their parent provided written consent. Consent and assent forms were mailed in advance of the first study visit and reviewed with all participants and parents, as applicable.

### Outcome measures

We adapted the MacArthur Competence Assessment Tool for Clinical Research, a structured interview to assess four aspects of decisional capacity: Understanding, Appreciation, Reasoning, and Making and Communicating a Choice [42].

#### Understanding

Thirteen multiple-choice items measured participants’ level of understanding about the clinical trial, such as what will happen when the participant sees the doctor (Cronbach’s alpha = 0.89). Participants were given two attempts to answer each question correctly; if a question was answered incorrectly, they were redirected to the information before answering the question again. Participants scored a 2 if they answered correctly on the first attempt, a 1 if they answered correctly on the second attempt, and 0 if they answered the question incorrectly on both attempts. We calculated a summary score, ranging from zero to 26, and translated scores into a percentile based on the number of questions answered. A score of 100% indicates that the participant answered each Understanding question correctly on the first attempt.

#### Appreciation

Three multiple-choice questions measured participants’ appreciation of how the clinical trial applies to them, such as why their doctor would tell them about the study (Cronbach’s alpha = 0.63). The same method of scoring was used for the Appreciation score as for the Understanding score.

#### Reasoning

For this component, participants were asked to provide two consequences for being in the study. The examiner assigned a Consequential Reasoning score of 0, 1, or 2 based on the number of logical consequences provided.

#### Expressing a choice

At the conclusion of the informed consent process, participants were asked whether they wanted to be in the study. Participants selected from “Yes,” “No,” or “I don’t know.”

### Independent measures

Other variables included as covariates were cognitive functioning, oral comprehension, verbal memory, and working memory. For these measures, higher scores indicate higher functioning/ability. Total general anxiety was also examined as a covariate, with higher scores indicating greater anxiety. Autism status was examined as an independent variable in simple comparisons.

#### Cognitive functioning

The *Stanford-Binet Intelligence Scales*, 5th edition, was used to measure cognitive functioning (IQ) [43]. The SB5 provides scores for verbal and nonverbal ability across five domains: Fluid Reasoning, Knowledge, Quantitative Reasoning, Visual-Spatial Reasoning, and Working Memory. Because standardized IQ tests have limited range and precision for those with ID, including people with FXS, we used a previously established method [41] of z-score transformation based on the norm sample from the SB5 to correct for floor effects. A score of 100 reflects the average score in a normative sample. An IQ score above 70 indicates no ID, 55–70 indicates mild ID, and 35–54 indicates moderate ID.

#### Oral comprehension

Oral comprehension refers to a participant’s ability to process and understand information delivered orally. One subtest (Test 3) from the *Woodcock-Johnson Tests of Achievement*, 3rd edition, was used to assess oral comprehension; a standardized score was used [44].

#### Verbal memory

Verbal memory refers to a participant’s ability to remember information delivered orally. A verbal memory index score was derived from the Story Memory and Verbal Learning subtests of the *Wide Range Assessment of Memory and Learning*, 2^nd^ edition [45].

#### Working memory

Working memory is a core domain of executive functioning and refers to a participant’s ability to retain new information and make it available for processing. The sum of scaled scores for the Working Memory subtest of the Stanford-Binet was used to assess working memory [43].

#### Co-occurring autism

The Social Communication Questionnaire Lifetime Form, traditionally a screening measure, was used as a measure of developmental history of autism spectrum disorder (ASD) based on caregiver report [46]. Additionally, the *Autism Diagnostic Observation Schedule*, 2^nd^ edition [47] (ADOS-2), which was administered by reliable assessors, was used as a direct assessment of ASD symptoms. After discussion among the clinical assessment team, participants who met criteria for ASD on both the SCQ and the ADOS-2 were considered to meet criteria for ASD for this study.

#### Anxiety

The *Anxiety*, *Depression*, *and Mood Scale* [48] is a parent-report questionnaire consisting of 28 items that serves as a screen for psychiatric disorders in individuals with ID. The scale’s psychometric properties were evaluated and normed with 265 individuals with ID and validated with a total of 129 psychiatric patients with ID [48]. One subscale was used as a measure of General Anxiety (seven items).

### Sample size

We conducted a power analysis using a between-subjects design (i.e., participants randomized to experimental or comparison group). With a sample size of 70 (35 participants per group), we had 90% power to detect an effect size of 0.75.

### Randomization

We used block randomization to assign participants to the experimental or comparison condition. Two stratification variables were used: Verbal IQ score (three levels) and age (two levels). Age was selected as a randomizing variable, because children and adolescents under 18 were not able to provide informed consent, only assent, as their parents are their legal guardians. Verbal IQ was selected as a second variable given the range of scores in the sample. Because enrollment for the RCT was done on a rolling basis, a 10-block, two-group design was used. We randomized 10 participants at a time using a random number generator (www.randomizer.com) to make the assignments.

### Blinding

Because of the nature of the study, participants and data collectors were not blinded to group assignment.

### Analyses

We conducted descriptive analyses to profile our sample and detect significant differences between the experimental and comparison participants. T-tests were used to examine differences in continuous variables. Chi-square tests were used to compare categorical variables; cells with small samples were collapsed (e.g., non-Hispanic White compared with all other races/ethnicities). We conducted all data analyses using SAS Enterprise Guide 7.1 software.

To answer research question 1, we examined the effect of the decision tool on Understanding, Appreciating, Reasoning, and Expressing a choice in unadjusted analyses and after controlling for sex, cognitive functioning (IQ), oral comprehension, verbal memory, working memory, and anxiety. Autism status was not included in the analysis due to risk for multicollinearity. For the continuous Understanding and Appreciation scores, we used t-tests to examine simple differences by experimental condition and linear regression in multivariate analyses. Because the Consequential Reasoning item is a three-level ordinal outcome, a Cochran Armitage trend test was used to examine for a simple differences by experimental condition, and ordinal logistic regression was used for multivariate analysis. The few participants who selected “Don’t know” for the Expressing a Choice item (n = 4) were removed from the analysis, making this a dichotomous item. Thus, chi-square tests were used in unadjusted analyses, whereas logistic regression was used in adjusted analysis. To ease interpretability, we standardized the covariates and the continuous outcome measures of Understanding and Appreciating for multivariate regressions.

For research question 2, we explored the impact of the decision support tool on individuals with higher levels of cognitive functioning (IQ). Participants who scored at or above the 25^th^ percentile for IQ (score of = 52) were in the “higher IQ” group (N = 66). We conducted several descriptive analyses to provide background information on our study population. First, we assessed if differences existed between those who were included in the higher IQ sample and those who were not. Next, within the higher IQ sample, we examined whether there were differences between the experimental and comparison groups \. Additionally, we used Cochran Armitage trend tests to test for item-level differences by experimental group for the Understanding domain among the higher IQ group. We reference the one-tailed test, because we tested whether the experimental condition would be related to significantly higher scores at the item level.

For research question 3, we used linear regressions to examine the effects of the experimental condition on decisional capacity measures with the Mac-CAT Understanding score as a predictor variable. This analysis was carried out in both the total sample and the higher IQ sample.

## Results

All participants from the larger study (N = 152) were assessed for eligibility. Forty-one participants were excluded: 39 did not meet the inclusion criteria, and 2 declined to participate in the follow-up study. Thus, 111 participants were randomized: 56 in the comparison group and 55 in the experimental group. One participant in the experimental group was scheduled but later declined. Twenty participants (11 in comparison group and 9 in experimental group) were lost to follow-up because of scheduling conflicts. One participant was given the intervention; however, due to technical difficulties with the tablet, these data were excluded. In total, there were 89 participants (44 experimental, 45 comparison) in the final analyses.

### Demographics of study participants

Participants were on average 21.2 years old (SD = 7.3; Range = 12.0–40.0 years); females in the full sample were between 12 and 39 years old (mean = 20.8); males were between the ages of 12 and 40 years old (mean = 21.9). Participants were mostly White (87.6%), female (58.4%), and without autism (80.9%; Table 1). Participants scored an average of 65.6 on the IQ measure (SD = 16.8); 38.2% had no ID, 29.2% had mild ID, and 32.6% had moderate ID. Most parents or caregivers were married (77.5%) and had at least a 4-year college degree (60.7%). Reported family incomes were relatively high. No significant differences existed between experimental and comparison groups for demographic or cognitive variables in the full sample (not shown). Within the experimental condition for the full sample, 62% of participants were female, compared to 46% of participants in the comparison group; this was a not statistically significant difference (*p* = 0.58).

**Table 1:** Characteristics of those in the full sample and higher IQ sample.

	Full Sample N = 89		Higher IQ Sample N = 66	
	N	Mean (SD)	N	Mean (SD)
Child’s age	89	21.2 (7.3)	66	20.3 (6.9)
Child’s IQ	88	65.6 (16.8)	66	72.1 (14.6)
Oral comprehension	89	83.3 (15.3)	66	86.1 (14.9)
Working memory	88	7.3 (6.1)	66	8.9 (6.2)
Verbal memory index	89	77.3 (15.2)	66	81.2 (14.5)
Total general anxiety	87	5.6 (3.9)	64	5.5 (3.8)
	**N**	**%**	**n**	**%**
Child’s sex				
Male	37	41.6	18	27.3
Female	52	58.4	48	72.7
Child’s race/ethnicity				
Non-Hispanic Black	3	3.4	2	3.0
Non-Hispanic White	78	87.6	60	90.9
Hawaiian/Pacific Islander	1	1.1	1	1.5
Hispanic/Latino	2	2.3	1	1.5
Multiple	1	1.1	1	1.5
Missing	4	4.5	1	1.5
Child’s autism status				
No	72	80.9	60	90.9
Yes	14	15.7	5	7.6
Missing	3	3.4	1	1.5
Family income category				
<$50,000	3	3.4	2	3.0
$50,001‒$75,000	7	7.9	6	9.0
$75,001‒ $100,000	14	15.7	10	15.2
>$100,000	22	24.7	15	22.7
Missing	43	48.3	33	50.0
Maternal education				
High school or less	4	4.5	1	1.5
Some college or associate degree	18	20.2	15	22.7
College degree	41	46.1	33	50.0
Master’s degree and above	13	14.6	9	13.6
Missing	13	14.6	8	12.1
Mother’s marital status				
Single, never married	6	6.7	4	6.1
Married	69	77.5	52	78.8
Divorced or widowed	9	10.1	7	10.6
Missing	5	5.6	3	4.6

Participants in the high IQ group were on average 20.3 years old (SD = 6.9; Range = 12.0–39.0 years), mostly female (72.7%), and without autism (90.9%; Table 1). Females in the high IQ group were between 12 and 39 years old (mean = 20.4); males were between the ages of 12 and 36 (mean = 19.8; S1 Table). In the subgroup analyses for research question 2, as expected, participants who were in the higher IQ group scored higher on measures of oral comprehension, working memory, and verbal memory (p’s < 0.01) and were less likely to have a co-diagnosis of autism (p < 0.001) than participants that were not included in additional analyses. Participants who were in the higher IQ group were also more likely to be female (p < 0.001) and slightly younger than those who were not allocated to that group (p = 0.03). No significant differences existed between experimental and comparison groups for demographic or cognitive variables in the high IQ sample (not shown). Within the high IQ group, 80% of participants in the experimental condition were female, compared to 67% of participants in the comparison group; this was a not statistically significant difference (*p* = 0.23).

### Comparison of the decision support tool with standard practice

Mean scores for participants in the experimental group were 74.4 (SD = 27.3) and 72.4 (SD = 27.5) on the domains of Understanding and Appreciating, respectively. Mean scores for participants in the comparison group were 75.1 (SD = 25.9) and 73.3 (SD = 32.1) on the domains of Understanding and Appreciating, respectively. Among the experimental group, 20 (45.5%) agreed they would participate in the clinical trial, and no one answered “Don’t know,” whereas 18 (40.0%) in the comparison group agreed to participate and 4 participants (8.9%) did not make a choice. Among the experimental group, 16 (40.0%) participants scored a 2 on the Consequential Reasoning item; 14 (33.3%) did so from the comparison group.

No significant differences emerged between participants in the experimental and comparison groups in the domains of Understanding or Appreciating in the unadjusted analyses (Understanding: t = 0.13, p = 0.89; Appreciating: t = 0.15, p = 0.88) or after controlling for sex, IQ, oral comprehension, verbal memory, working memory, and total anxiety (Understanding: *β* = 0.16, p = 0.27; Appreciating: *β* = 0.08, p = 0.64; Table 2). No significant differences existed between the experimental and comparison groups in the Reasoning or Expressing a Choice domains in the unadjusted (Consequential Reasoning: *Z* = -0.01, *p* = 0.99; Expressing a Choice: χ^2^ = 0.02, *p* = 0.89) or adjusted analyses (Consequential Reasoning: *OR* = 1.63, *p* = 0.32; Expressing a Choice: *OR* = 1.10, *p* = 0.84). Full scale IQ (*β* = 0.71, *p* = 0.002) and verbal memory (*β* = 0.24, *p* = 0.046) were significant predictors of the Understanding domain, whereas only oral comprehension significantly predicted the Appreciation (*β* = 0.29, *p* = 0.02) and Reasoning domains (*OR* = 2.62, *p* = 0.02).

**Table 2:** Effects of experimental condition on decisional capacity measures in the full sample.

	Understanding^a^ *β (95% CI)*	Appreciating^a^ *β (95% CI)*	Expressing a Choice^b^ *OR (95% CI)*	Consequential Reasoning^c^ *OR (95% CI)*
N	84	83	82	80
Experimental condition	0.16 (−0.13, 0.44)	0.08 (−0.26, 0.42)	1.10 (0.44, 2.78)	1.63 (0.63, 4.26)
Sex (female)	0.03 (-0.36, 0.42)	0.21 (-0.25, 0.67)	1.59 (0.45, 5.60)	2.54 (0.69, 9.42)
IQ	0.71 (0.27, 1.15)**	0.45 (-0.07, 0.96) †	0.74 (0.19, 2.95)	2.91 (0.68, 12.54)
Oral comprehension	0.20 (−0.01, 0.41) †	0.29 (0.04, 0.53)*	0.77 (0.40, 1.50)	2.62 (1.17, 5.89)*
Verbal memory	0.24 (0.01, 0.48)*	0.02 (−0.26, 0.30)	1.23 (0.57, 2.66)	1.52 (0.71, 3.26)
Working memory	−0.33 (−0.71, 0.06) †	-0.07 (−0.52, 0.39)	1.74 (0.50, 6.04)	0.48 (0.12, 1.89)
Total General Anxiety	-0.13 (-0.27, 0.01) †	-0.14 (-0.32, 0.03) †	1.14 (0.71, 1.82)	0.97 (0.59, 1.61)

^†^p < 0.10

*p < 0.05

**p < 0.01.

^a^Linear regression.

^b^Logistic regression.

^c^Ordinal logistic regression.

In secondary analyses, we examined to what extent the Understanding domain predicted Appreciating, Expressing a Choice, or Reasoning (see Table 3). Understanding was positively associated with the Appreciating (*β* = 0.63, *p <* 0.001) domain; oral comprehension was no longer related to Appreciation when Understanding was in the model.

**Table 3:** Effects of experimental condition on decisional capacity measures in the full sample with MacCAT Understanding as a predictor.

	Appreciating^a^ *β (95% CI)*	Expressing a Choice^b^ *OR (95% CI)*	Consequential Reasoning^c^ *OR (95% CI)*
N	83	80	78
Experimental condition	−0.04 (−0.33, 0.25)	1.05 (0.41, 2.70)	1.22 (0.45, 3.31)
Understanding	0.63 (0.39, 0.86)**	1.15 (0.54, 2.47)	2.10 (0.86, 5.09)
Female sex	0.20 (-0.20, 0.60)	1.55 (0.44, 5.51)	3.07 (0.78, 12.14)
IQ	0.03 (−0.44, 0.50)	0.59 (0.13, 2.67)	1.43 (0.28, 7.35)
Oral comprehension	0.16 (−0.06, 0.37)	0.75 (0.38, 1.50)	2.09 (0.90, 4.86) †
Verbal memory	−0.12 (−0.37, 0.13)	1.18 (0.53, 2.62)	1.54 (0.69, 3.41)
Working memory	0.12 (−0.28, 0.51)	2.02 (0.56, 7.38)	0.67 (0.16, 2.75)
Total General Anxiety	-0.09 (-0.24, 0.06)	1.13 (0.69, 1.84)	1.04 (0.61, 1.77)

^†^p < 0.10

*p < 0.05

**p < 0.01

^a^Linear regression.

^b^Logistic regression.

^c^Ordinal logistic regression.

### Benefits of the decision support tool for those with higher cognitive functioning

Among the higher IQ subsample, participants scored a mean of 86.3 (SD = 15.3) and 80.8 (SD = 22.2) on the domain of Understanding in the experimental and comparison groups, respectively. There was no difference in Understanding in unadjusted analyses (*T* = -1.15, *p* = 0.25); however, participants in the experimental condition achieved higher Understanding scores after adjusting for covariates (*β* = 0.25, *p* = 0.04; Table 4). Oral comprehension (*β* = 0.26, *p* = 0.006) and verbal memory (*β* = 0.19, *p* = 0.05) were also significantly associated with Understanding in the multiple linear regression model.

**Table 4:** Effects of experimental condition on decisional capacity measures in the higher IQ sample.

	Understanding^a^ *β (95% CI)*	Appreciating^a^ *β (95% CI)*	Expressing a Choice^b^ *OR (95% CI)*	Consequential Reasoning^c^ *OR (95% CI)*
N	63	63	60	59
Experimental condition	0.25 (0.01, 0.49)*	0.14 (-0.23, 0.51)	1.01 (0.34, 3.00)	1.65 (0.56, 4.87)
Sex (female)	0.24 (-0.10, 0.57)	0.13 (-0.39, 0.64)	2.22 (0.50, 9.99)	4.51 (0.98, 20.85) †
IQ	0.30 (-0.19, 0.77)	0.58 (-0.16, 1.32)	0.54 (0.07, 4.23)	1.34 (0.17, 10.48)
Oral comprehension	0.26 (0.08, 0.44)**	0.32 (0.04, 0.60)*	0.94 (0.42, 2.12)	1.82 (0.75, 4.42)
Verbal memory	0.19 (0.01, 0.39)*	0.04 (−0.26, 0.33)	1.02 (0.43, 2.42)	1.23 (0.53, 2.87)
Working memory	−0.11 (−0.48, 0.26)	−0.21 (−0.79, 0.36)	1.96 (0.39, 9.87)	1.04 (0.19, 5.64)
Total General Anxiety	-0.11 (-0.24, 0.01) †	-0.17 (-0.36, 0.03) †	1.04 (0.59, 1.84)	1.09 (0.60, 1.97)

^†^p < 0.10

*p < 0.05

**p < 0.01

^a^Linear regression.

^b^Logistic regression.

^c^Ordinal logistic regression.

In the higher IQ subsample, participants in the experimental group scored a mean of 81.0 (SD = 23.5) and 78.7 (SD = 30.0) on the domain of Appreciating in the experimental and comparison groups, respectively. There was no significant difference in the Appreciation score between experimental and comparison conditions in the unadjusted analyses (*T* = -0.34, p = 0.73) nor after controlling for the cognitive variables (Table 4, *β* = 0.14, p = 0.45). Oral comprehension (*β* = 0.32, p = 0.03) remained the only significant related to the Appreciating domain in the adjusted model within the higher IQ sample.

Fifteen participants in each of the experimental (50.0%) and comparison groups (41.7%) agreed to participate in the hypothetical clinical trial; in the comparison condition, 4 participants (11.1%) answered “Don’t know.” The experimental condition was unrelated to the participants’ decision to join the trial in the unadjusted (*χ*^*2*^ = 0.06, *p* = 0.81) and adjusted analyses (*OR* = 1.01, *p* = 0.99).

In the higher IQ sample, 15 participants (55.6%) in the experimental group nominated two logical consequences; 13 participants (38.3%) did so from the comparison group. There was no difference between the experimental and comparison groups in the number of consequences given in the unadjusted (*Z* = -0.91, p = 0.36) nor adjusted analyses (Table 4, *OR* = 1.65, *p* = 0.37).

In secondary analyses in which we additionally examined Understanding as a predictor of other decisional capacity domains, higher Understanding was significantly associated with greater Appreciating (Table 5; *β* = 0.66, *p* = 0.001) only. Oral comprehension and verbal memory were also not longer related to Appreciating when Understanding was included in the model.

**Table 5:** Effects of experimental condition on decisional capacity measures in the higher IQ sample with MacCAT Understanding as a predictor.

	Appreciating^a^ *β (95% CI)*	Expressing a Choice^b^ *OR (95% CI)*	Consequential Reasoning^c^ *OR (95% CI)*
N	63	59	58
Experimental condition	-0.03 (−0.38, 0.33)	0.79 (0.25, 2.53)	1.41 (0.44, 4.90)
Understanding	0.66 (0.28, 1.04)**	1.85 (0.51, 6.69)	1.40 (0.39, 4.95)
Sex (female)	-0.03 (-0.51, 0.45)	2.08 (0.44, 9.73)	4.26 (0.90, 20.18)
IQ	0.38 (−0.30, 1.07)	0.33 (0.04, 2.93)	0.98 (0.11, 8.59)
Oral comprehension	0.15 (−0.13, 0.43)	0.79 (0.33, 1.90)	1.63 (0.64, 4.12)
Verbal memory	-0.09 (−0.37, 0.19)	0.92 (0.37, 2.30)	1.19 (0.50, 2.85)
Working memory	−0.14 (−0.67, 0.39)	2.65 (0.49, 14.32)	1.24 (0.22, 7.03)
Total General Anxiety	-0.09 (-0.27, 0.09)	1.09 (0.59, 2.00)	1.10 (0.60, 2.04)

^†^p < 0.10

*p < 0.05

**p < 0.01

^a^Linear regression.

^b^Logistic regression.

^c^Ordinal logistic regression.

### Understanding specific components of the trial

Regarding individual items used to create the Understanding score within the higher IQ sample, participants in the experimental condition were more likely to answer correctly “Why are the doctors doing the study” (*p* = 0.009), “How often will you need to take the medicine?” (*p* = 0.04, “What is one thing you will do at the doctor’s office?” (*p* = 0.01), and “What is another thing you will do when you see the doctor?” (*p* = 0.03; Table 6).

**Table 6:** Comparisons of experimental and comparison conditions on item-level understanding among participants in the higher IQ sample.

Multiple-choice question	Scoring	Comparison (n = 36) N (%)	Experimental (n = 30) N (%)	1-sided P-value
1. What is the study about? a. Candy b. Dr. Bell c. Lots of people **d. A medicine to help people with fragile X**	0‒Never correct	1 (2.8)	2 (6.9)	0.22
	1‒Correct on 2nd try	0 (0.0)	0 (0.0)	
	2‒Correct on 1st try	35 (97.2)	27 (93.1)	
2. Why are the doctors doing the study? **a. To test if the medicine will help with fragile X** b. To make money c. To help Alex run faster d. To see if the answers are correct	0‒Never correct	2 (5.6)	0 (0.0)	0.009
	1‒Correct on 2nd try	5 (13.9)	0 (0.0)	
	2‒Correct on 1st try	29 (80.6)	29 (100.0)	
3. How often will you need to take the medicine? a. Three times a day b. When I am hungry **c. Two times a day** d. Only when I eat breakfast	0‒Never correct	10 (27.8)	1 (3.5)	0.04
	1‒Correct on 2nd try	3 (8.3)	7 (24.1)	
	2‒Correct on 1st try	23 (63.9)	21 (72.4)	
4. What is one thing you will do at the doctor’s office? a. Eat lunch **b. Pee in a cup** c. Get my ears checked d. Get a shot	0‒Never correct	6 (16.7)	1 (3.5)	0.01
	1‒Correct on 2nd try	4 (11.1)	0 (0.0)	
	2‒Correct on 1st try	26 (72.2)	28 (96.6)	
5. What is another thing you will do when you see the doctor? **a. Answer questions about how I feel** b. Have my temperature checked c. Brush my teeth d. Take a math test	0‒Never correct	3 (8.3)	0 (0.0)	0.01
	1‒Correct on 2nd try	5 (13.9)	1 (3.5)	
	2‒Correct on 1st try	28 (77.8)	28 (96.5)	
6. Why are there two types of pill—a real pill and a fake pill? a. So I have one with breakfast and one with dinner b. So everyone gets the real pill **c. So the doctors can learn if the real pill is better than the fake pill** d. So that I can have one and my doctor can have one.	0‒Never correct	7 (19.4)	9 (31.0)	0.31
	1‒Correct on 2nd try	7 (19.4)	2 (6.9)	
	2‒Correct on 1st try	22 (61.1)	18 (62.1)	
7. How will it be decided which pill you will take? a. I will decide **b. A computer will decide** c. My parents will decide d. My doctor will decide	0‒Never correct	10 (27.8)	6 (20.7)	0.14
	1‒Correct on 2nd try	7 (19.4)	3 (10.3)	
	2‒Correct on 1st try	19 (52.8)	20 (69.0)	
8. Who will know which pill you are taking? a. My doctor will know **b. No one will know** c. My parents will know d. I will know	0‒Never correct	11 (30.6)	8 (27.6)	0.42
	1‒Correct on 2nd try	5 (13.9)	7 (24.1)	
	2‒Correct on 1st try	20 (55.6)	14 (48.3)	
9. What will the doctors learn from the study? a. If I am happy b. If I like my pills c. If my heart is healthy **d. If the medicine helps lots of people with fragile X**	0‒Never correct	1 (2.8)	0 (0.0)	0.11
	1‒Correct on 2nd try	3 (8.3)	1 (3.5)	
	2‒Correct on 1st try	32 (88.9)	28 (96.5)	
10. What is one good thing that might happen to you if you are in the study? a. I might get to choose the real pill b. I might lose weight **c. I might feel calmer or pay attention easier** d. I might win a prize	0‒Never correct	5 (13.9)	2 (6.9)	0.13
	1‒Correct on 2nd try	4 (11.1)	2 (6.9)	
	2‒Correct on 1st try	27 (75.0)	25 (86.2)	
11. What is one thing that you might not like if you are part of the study? **a. I might feel a little sick or tired** b. I might get a rash c. I might miss my favorite show on TV d. I might have to play on the computer	0‒Never correct	5 (13.9)	2 (6.9)	0.12
	1‒Correct on 2nd try	3 (8.3)	1 (3.5)	
	2‒Correct on 1st try	28 (77.8)	26 (89.7)	
12. What is one thing that would happen to you that might hurt? a. I will have to do a lot of jumping jacks b. I will get my ears checked c. I will have to talk to the doctor **d. I will have my blood drawn**	0‒Never correct	2 (5.6)	2 (6.9)	0.10
	1 ‒ Correct on 2nd try	0 (0.0)	4 (13.8)	
	2 ‒ Correct on 1st try	34 (94.4)	23 (79.3)	
13. What should you do if you do not want to be in the study? a. Tell the computer not to pick me b. Find a different doctor **c. Tell the doctor I don’t want to be in the study** d. Say yes and be in the study anyway	0 ‒ Never correct	3 (8.3)	4 (13.8)	0.29
	1 ‒ Correct on 2nd try	2 (5.6)	1 (3.5)	
	2 ‒ Correct on 1st try	31 (86.1)	17 (82.8)	

See S2, S3 and S4 Tables for the above results within the higher IQ subsample.

### Summary of findings

All research studies, including clinical trials, must adhere to federal guidelines and ethical principles of informed consent. Particularly for vulnerable populations, including those with ID, informed consent is critical both for reasons of science (including the need to demonstrate broad effectiveness) and distributive justice (to ensure that vulnerable individuals have equal access to potentially beneficial treatments) [49]. The goal of this paper was to compare the efficacy of a digital decision support tool with standard practice for informed consent among individuals with FXS and to examine whether the tool can improve decisional capacity for higher functioning individuals.

The tool was not associated with greater levels of understanding and appreciation for the full sample of individuals with FXS. There may be a threshold below which individuals with ID do not benefit from support as a result of the severity of their impairment and must rely on proxy decision makers. The tool was associated with greater understanding of the clinical trial among higher cognitive functioning individuals. Participants in the higher IQ group were characterized by higher cognitive abilities, including oral comprehension, working memory, and verbal memory. These findings suggest that decision support tools may benefit participants with the higher range of moderate impairments, mild, or no cognitive impairments and do so without undue influence on their decision whether to participate in the trial. Prior research has also found no impact of audio-visual decision supports on participants’ decision to join the trial [20]. The FXS subpopulation with mild cognitive impairment could be targeted for future development of decision supports, potentially along with other clinical populations with mild cognitive impairment.

Among the full sample, and among the higher IQ subsample specifically, understanding (i.e., perceiving and retaining information related to the decision to join the clinical trial) was a strong predictor of appreciation (i.e., ones’ ability to link the decision to one’s own situation). This is consistent with our previous research [38] using an expanded sample of participants from this study, and with the results of a study by Fisher et al. [25], which found that capacity to consent depends on the ability of the information to be understood by participants. These findings support the need for consent materials to be delivered in a way that augments participant understanding, thus maximizing participants’ ability to make a personal, informed decision based on their own circumstances, beliefs, and wishes. The decision aid addresses the gap between how informed consent is currently conceptualized and operationalized. As an established measurement approach, the MacCAT can be used to assess whether the operationalization of consent using the decision aid is equal to or better than the standard paper form.

Higher functioning study participants within the experimental condition were more likely to understand concrete elements of the trial namely their responsibilities as a participant including what they are being asked to do (i.e. how many times per day they will have to take medicine) and what they would need to do in the doctor’s office (i.e., have their blood drawn). This is consistent with findings from our earlier research using paper-based materials with this study population [38]. While high quality evidence is lacking about the impact of audio-visual interventions on the consent process, some hypothesize that it may help individuals reduce anxiety associated with the experience [20]; this may be an indirect benefit associated with increased knowledge, greater appreciation and satisfaction with the information provided. The risk communication literature has shown that visual aids can help people comprehend quantitative efficacy and risk information, but not all visual aids are effective [50].

Our earlier work also demonstrated the importance of repeating information and using alternative ways to convey knowledge, suggesting that scaffolding can improve retention and ultimately understanding [38]. Although the quality of the existing evidence is low, according to a systematic review of audio-visuals that informed consent interventions, the overall body of evidence suggests that an audio-visual strategy may slightly improve knowledge levels [20], which is the case with our findings. The human-like avatar who engages the user and promotes bidirectional communication may be a key factor in helping with understanding complex information, distinguishing it from other multimedia tools. The tool also employed best practices in communication and decision science that recommend presenting information using shortened and user-friendly content [51] and strategies for the presentation of probability data [52].

### Limitations and future directions

The study is not without limitations. First, it involved a hypothetical trial. Second, examiners were not blinded to the study condition, introducing the possibility of bias. The study had a relatively small sample size, and we used participants’ IQs as the sole measure to define groups by level of cognitive functioning. This small sample size limited our ability to explore for experimental effects by participant characteristics such as comorbidities including anxiety disorder, attention-deficit hyperactive disorder (ADHD), or ASD. Future studies may benefit from oversampling individuals with comorbidities in order to investigate for differential effects. Considering the distinct sex-dependent phenotypes of fragile X syndrome, additional research may explore if the tool may differentially benefit individuals with ID based on their sex. Future studies should also examine the level of cognitive capacity a potential participant would need to have sufficient understanding to provide informed consent. Is there a certain threshold at which an individual is able to independently make a decision with appropriate supports? The effect of the decision support tool should be examined in larger-scale studies in the context of actual clinical trials to assess the impact on consent, trial retention, and cost-effectiveness. Finally, although we focused on individuals with FXS, the decision support tool may provide benefit for those with other forms of ID or those from the general population who need additional supports (e.g., low health literacy) to fully participate in the informed consent process. We recognize that digital decision support tools may be limited in what they can achieve and that other methods of support should be explored as well. Nonetheless, the use of an interactive decision support tool showed promise for individuals with no ID, mild ID, and higher levels of moderate ID. It may prove valuable for populations without cognitive impairment who are overwhelmed by the current approach to informed consent.

## Conclusions

This type of decision support tool offers advantages in a clinical setting for busy practitioners and patients, including customizing content and literacy level to the needs of the study population and presenting it as a supplement to conversations with human clinicians. Standardizing the presentation of content to all study participants promotes equitable and systematic dissemination of information. Knowledge assessment questions embedded throughout the consent process seek to increase interactivity and confirm subject comprehension. They objectively measure understanding versus only assessing recall of information or a subjective self-assessment of one’s understanding. The tool also enables adherence to recent changes in the Common Rule and promotes communication between patients and clinicians in support of shared decision making. Improved levels of understanding about key methodological procedures and trial responsibilities may lead to greater adherence to the protocol, longer-term retention, and higher quality data.

## Supporting information

S1 Table Characteristics of those in the full sample and higher IQ sample by child’s sex. (DOCX)

S2 Table Comparisons of experimental and comparison conditions on item-level appreciation among participants in the higher IQ sample. (DOCX)

S3 Table Comparisons of experimental and comparison conditions on item-level expressing a choice among participants in the higher IQ sample. (DOCX)

S4 Table Comparisons of experimental and comparison conditions on item-level consequential reasoning among participants in the higher IQ sample. (DOCX)
