# Supplementary material for: Supporting informed clinical trial decisions: Results from a randomized controlled trial evaluating a digital decision support tool for those with intellectual disability
Source: PLoS One. 2019 Oct 23;14(10):e0223801. doi: 10.1371/journal.pone.0223801 (PMC6808417; doi:10.1371/journal.pone.0223801)
Supplement: S1 Table — (DOCX) [file pone.0223801.s001.docx]

**S1: Characteristics of those in the full sample and higher IQ sample by child’s sex.**

|  | **Full Sample** | | | | **High IQ Sample** | | | |
| --- | --- | --- | --- | --- | --- | --- | --- | --- |
|  | **Males N = 37** | | **Females N = 52** | | **Males N = 18** | | **Females N = 48** | |
|  | **N** | **Mean (SD)** | **N** | **Mean (SD)** | **N** | **Mean (SD)** | **N** | **Mean (SD)** |
| Child’s age | 37 | 21.9 (7.5) | 52 | 20.8 (7.1) | 18 | 19.8 (6.7) | 48 | 20.4 (7.0) |
| Child’s IQ | 37 | 45.5 (7.5) | 51 | 67.4 (17.6) | 18 | 59.8 (7.3) | 48 | 76.7 (13.4) |
| Oral comprehension | 37 | 76.6 (14.2) | 52 | 88.2 (14.3) | 18 | 77.3 (16.7) | 48 | 89.4 (12.9) |
| Working memory | 37 | 3.5 (2.9) | 51 | 10.1 (6.4) | 18 | 4.7 (3.6) | 48 | 10.5 (6.3) |
| Verbal memory index | 37 | 68.2 (10.7) | 52 | 83.3 (14.8) | 18 | 71.5 (11.0) | 48 | 84.8 (14.1) |
| Total anxiety | 37 | 5.9 (3.9) | 50 | 5.3 (4.0) | 18 | 6.0 (3.2) | 46 | 5.3 (4.0) |
|  | **N** | **%** | **N** | **%** | **N** | **%** | **N** | **%** |
| Child’s race/ethnicity |  |  |  |  |  |  |  |  |
| Non-Hispanic Black | 1 | 2.7 | 2 | 3.9 | 0 | 0.0 | 2 | 4.2 |
| Non-Hispanic White | 32 | 86.5 | 46 | 88.5 | 17 | 94.4 | 43 | 89.6 |
| Hawaiian/Pacific Islander | 0 | 0.0 | 1 | 1.9 | 0 | 0.0 | 1 | 2.1 |
| Hispanic/Latino | 1 | 2.7 | 1 | 1.9 | 1 | 5.6 | 0 | 0.0 |
| Multiple | 0 | 0.0 | 1 | 1.9 | 0 | 0.0 | 1 | 2.1 |
| Missing | 3 | 8.1 | 1 | 1.9 | 0 | 0.0 | 1 | 2.1 |
| Child’s autism status |  |  |  |  |  |  |  |  |
| No | 25 | 67.6 | 47 | 90.4 | 15 | 83.3 | 45 | 93.8 |
| Yes | 11 | 29.7 | 3 | 5.8 | 2 | 11.1 | 3 | 6.3 |
| Missing | 1 | 2.7 | 2 | 3.9 | 1 | 5.6 | 0 | 0.0 |
| Family income category |  |  |  |  |  |  |  |  |
| <$50,000 | 1 | 2.7 | 2 | 3.9 | 0 | 0.0 | 2 | 4.2 |
| $50,001‒$75,000 | 4 | 10.8 | 3 | 5.8 | 3 | 16.7 | 3 | 6.3 |
| $75,001‒ $100,000 | 6 | 16.2 | 8 | 15.4 | 2 | 11.1 | 8 | 16.7 |
| >$100,000 | 11 | 29.7 | 11 | 21.2 | 6 | 33.3 | 9 | 18.8 |
| Missing | 15 | 40.5 | 28 | 53.9 | 7 | 38.9 | 26 | 54.2 |
| Maternal education |  |  |  |  |  |  |  |  |
| High school or less | 2 | 5.4 | 2 | 3.9 | 0 | 0.0 | 1 | 2.1 |
| Some college or associate degree | 6 | 16.2 | 12 | 23.1 | 4 | 22.2 | 11 | 22.9 |
| College degree | 16 | 43.2 | 25 | 48.1 | 10 | 55.6 | 23 | 47.9 |
| Master’s degree and above | 7 | 18.9 | 6 | 11.5 | 3 | 16.7 | 6 | 12.5 |
| Missing | 6 | 16.2 | 7 | 13.5 | 1 | 5.6 | 7 | 14.6 |
| Mother’s marital status |  |  |  |  |  |  |  |  |
| Single, never married | 2 | 5.4 | 4 | 7.7 | 0 | 0.0 | 4 | 8.3 |
| Married | 31 | 83.8 | 38 | 73.1 | 17 | 94.4 | 35 | 72.9 |
| Divorced or widowed | 1 | 2.7 | 8 | 15.4 | 0 | 0.0 | 7 | 14.6 |
| Missing | 3 | 8.1 | 2 | 3.9 | 1 | 5.6 | 2 | 4.2 |
