# Supplementary material for: Supporting informed clinical trial decisions: Results from a randomized controlled trial evaluating a digital decision support tool for those with intellectual disability
Source: PLoS One. 2019 Oct 23;14(10):e0223801. doi: 10.1371/journal.pone.0223801 (PMC6808417; doi:10.1371/journal.pone.0223801)
Supplement: S2 Table — (DOCX) [file pone.0223801.s002.docx]

**S2. Comparisons of experimental and comparison conditions on item-level appreciation among participants in the higher IQ sample.**

| **Multiple-choice question** | **Scoring** | **Comparison (n = 36) N (%)** | **Experimental (n = 30) N (%)** | **1-sided P-value** |
| --- | --- | --- | --- | --- |
| 1. Why would your doctor tell you about the study?   a. Because the doctor likes me  **b. Because I have fragile X**  c. Because I have a cold  d. Because I am old enough | 0‒Never correct | 4 (11.1) | 0 (0.0) | 0.05 |
|  | 1‒Correct on 2nd try | 2 (5.6) | 2 (6.9) |  |
|  | 2‒Correct on 1st try | 30 (83.3) | 27 (93.1) |  |
| 2. Will you get the real pill or the fake pill?  **a. I do not know which pill I will get.**  b. I will get the fake pill because I don’t need the real pill.  c. I will get the real pill because I want to feel better.  d. I will get both pills. | 0‒Never correct | 8 (22.2) | 8 (28.6) | 0.33 |
|  | 1‒Correct on 2nd try | 5 (13.9) | 3 (10.7) |  |
|  | 2‒Correct on 1st try | 23 (63.9) | 17 (60.7) |  |
| 3. What will happen if you decide you no longer want to be in the study?  a. Once I join, I have to stay in the study.  b. I can stop and restart anytime I want.  **c. I will tell my doctor I want to stop and then can stop.**  d. My doctor will be angry. | 0‒Never correct | 5 (13.9) | 3 (10.3) | 0.49 |
|  | 1‒Correct on 2nd try | 5 (13.9) | 6 (20.7) |  |
|  | 2‒Correct on 1st try | 26 (72.2) | 20 (69.0) |  |
