# Supplementary material for: Supporting informed clinical trial decisions: Results from a randomized controlled trial evaluating a digital decision support tool for those with intellectual disability
Source: PLoS One. 2019 Oct 23;14(10):e0223801. doi: 10.1371/journal.pone.0223801 (PMC6808417; doi:10.1371/journal.pone.0223801)
Supplement: S3 Table — (DOCX) [file pone.0223801.s003.docx]

**S3. Comparisons of experimental and comparison conditions on item-level expressing a choice item among participants in the higher IQ sample.**

| **Open-ended question** | **Scoring** | **Comparison**  **(n = 34) N (%)** | **Experimental**  **(n = 27) N (%)** | **P-value** |
| --- | --- | --- | --- | --- |
| Why do you think it is better for you to be in the study than to not be in the study?  OR  Why do you think it is better for you to NOT be in the study than to be in the study? | 0 Logical Consequences | 8 (23.5) | 6 (22.2) | 0.36 |
|  | 1 Logical Consequences | 13 (38.2) | 6 (22.2) |  |
|  | 2 Logical Consequences | 13 (38.2) | 15 (55.6) |  |
