# Supplementary material for: Supporting informed clinical trial decisions: Results from a randomized controlled trial evaluating a digital decision support tool for those with intellectual disability
Source: PLoS One. 2019 Oct 23;14(10):e0223801. doi: 10.1371/journal.pone.0223801 (PMC6808417; doi:10.1371/journal.pone.0223801)
Supplement: S4 Table — (DOCX) [file pone.0223801.s004.docx]

**S4. Comparisons of experimental and comparison conditions on item-level consequential reasoning among participants in the higher IQ sample.**

| **Open-ended question** | **Scoring** | **Comparison**  **(n = 36) N (%)** | **Experimental**  **(n = 30) N (%)** | **1-sided P-value** |
| --- | --- | --- | --- | --- |
| Now that you have had a chance to think things through, would you like to be in the study or not be in the study? | Yes, I want to be in the study | 15 (41.7) | 15 (50.0) | 0.16 |
|  | No, I don’t want to be in the study | 17 (47.2) | 15 (50.0) |  |
|  | I’m not sure or don’t know | 4 (11.1) | 0 (0.0) |  |
